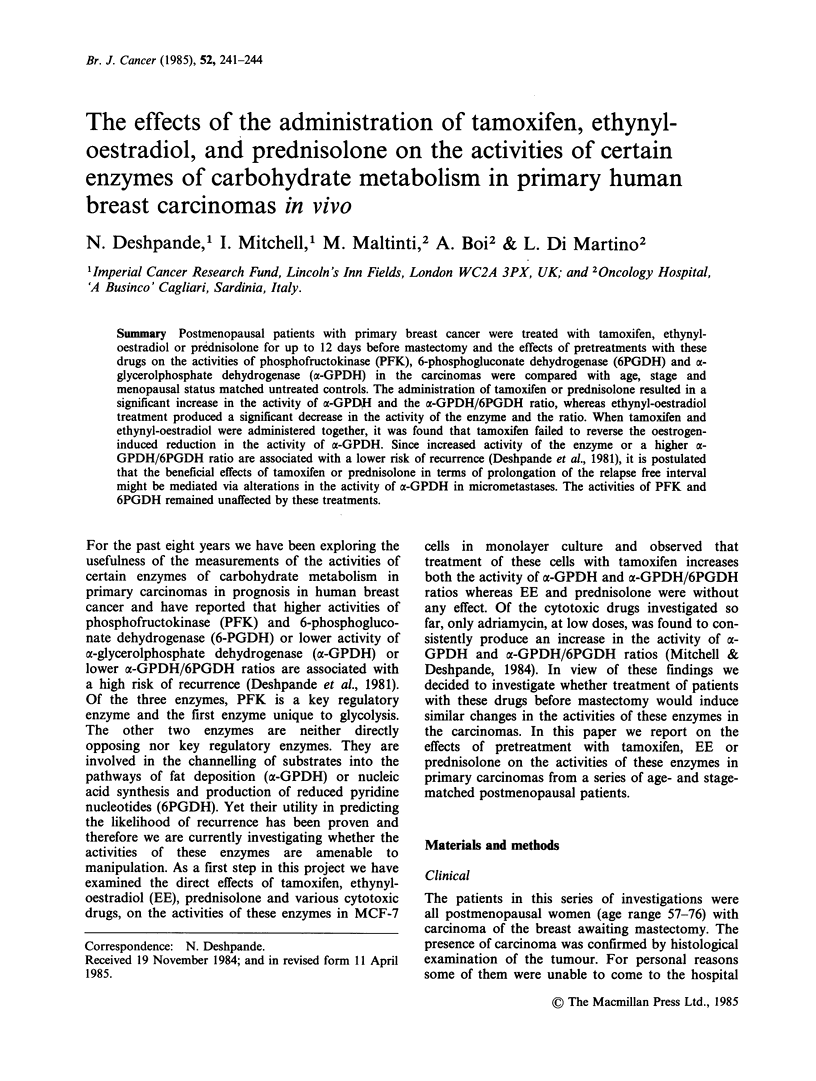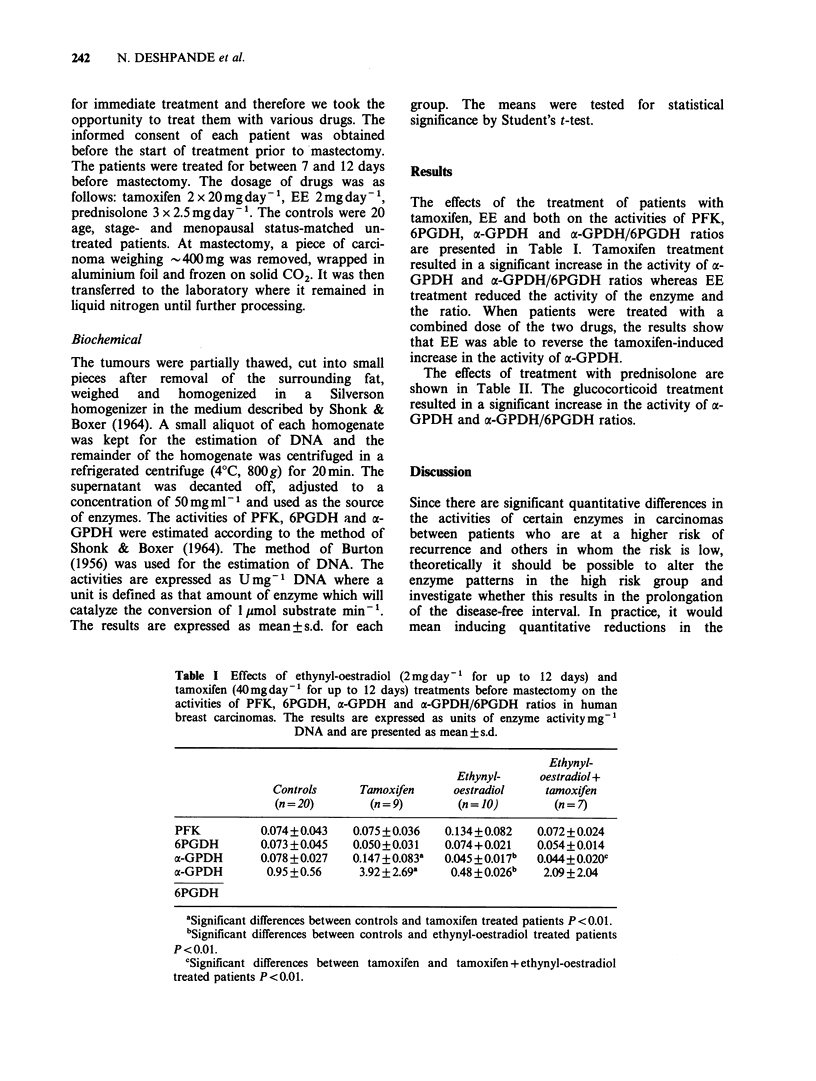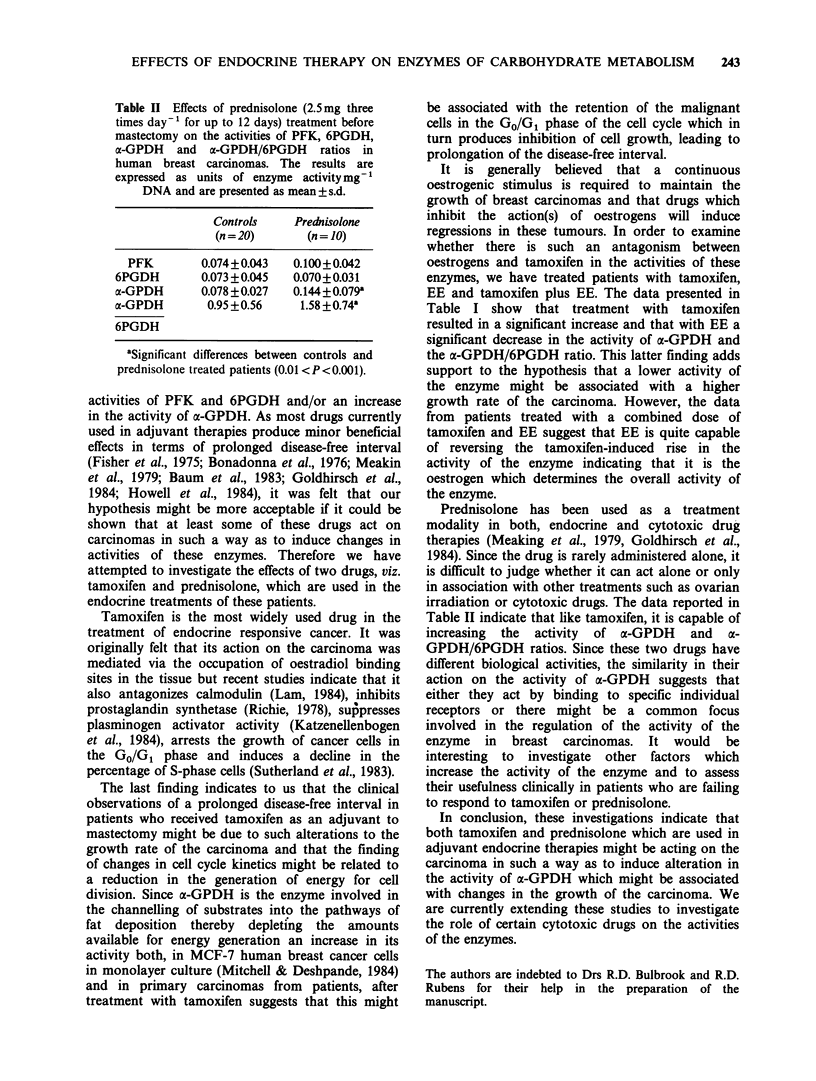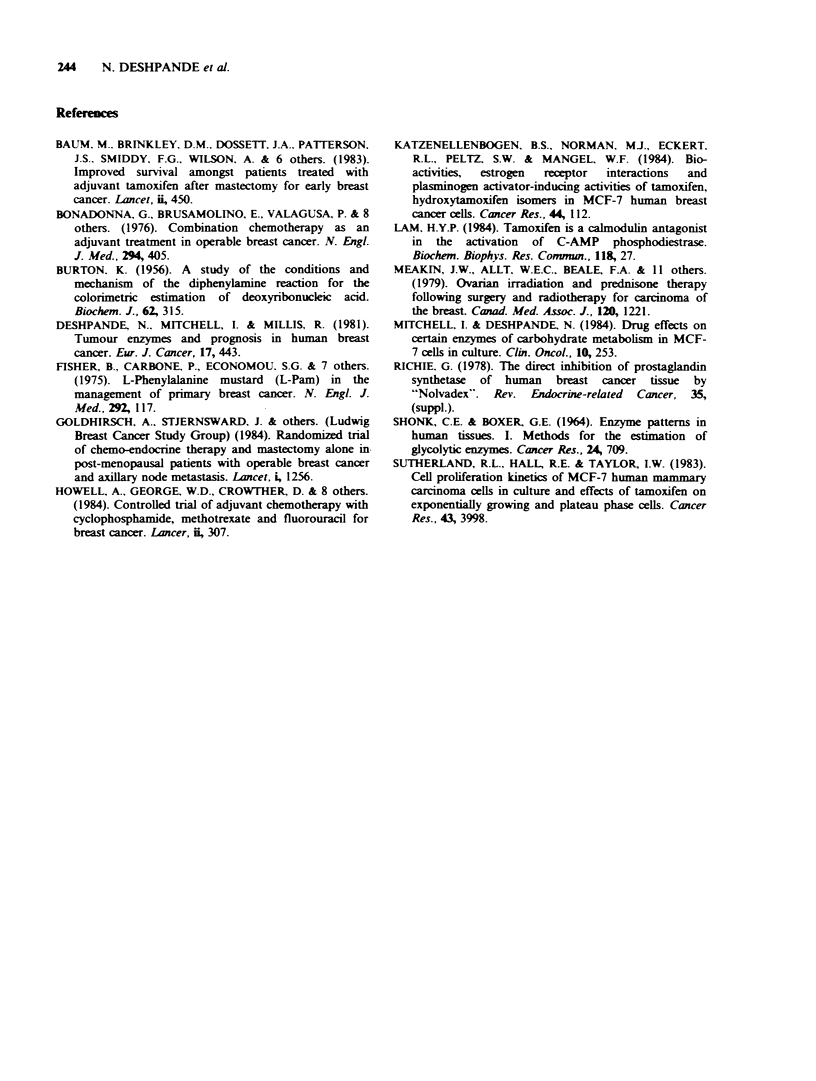# The effects of the administration of tamoxifen, ethynyloestradiol, and prednisolone on the activities of certain enzymes of carbohydrate metabolism in primary human breast carcinomas in vivo.

**DOI:** 10.1038/bjc.1985.183

**Published:** 1985-08

**Authors:** N. Deshpande, I. Mitchell, M. Maltinti, A. Boi, L. Di Martino

## Abstract

Postmenopausal patients with primary breast cancer were treated with tamoxifen, ethynyloestradiol or prednisolone for up to 12 days before mastectomy and the effects of pretreatments with these drugs on the activities of phosphofructokinase (PFK), 6-phosphogluconate dehydrogenase (6PGDH) and alpha-glycerolphosphate dehydrogenase (alpha-GPDH) in the carcinomas were compared with age, stage and menopausal status matched untreated controls. The administration of tamoxifen or prednisolone resulted in a significant increase in the activity of alpha-GPDH and the alpha-GPDH/6PGDH ratio, whereas ethynyl-oestradiol treatment produced a significant decrease in the activity of the enzyme and the ratio. When tamoxifen and ethynyl-oestradiol were administered together, it was found that tamoxifen failed to reverse the oestrogen-induced reduction in the activity of alpha-GPDH. Since increased activity of the enzyme or a higher alpha-GPDH/6PGDH ratio are associated with a lower risk of recurrence (Deshpande et al., 1981), it is postulated that the beneficial effects of tamoxifen or prednisolone in terms of prolongation of the relapse free interval might be mediated via alterations in the activity of alpha-GPDH in micrometastases. The activities of PFK and 6PGDH remained unaffected by these treatments.


*Br. J. Cancer* (1985), **52**, 241–244

### The effects of the administration of tamoxifen, ethynyl-oestradiol, and prednisolone on the activities of certain enzymes of carbohydrate metabolism in primary human breast carcinomas *in vivo*

N. Deshpande,[1] I. Mitchell,[1] M. Maltinti,[2] A. Boi[2] & L. Di Martino[2]

[1]*Imperial Cancer Research Fund, Lincoln's Inn Fields, London WC2A 3PX, UK; and* [2]*Oncology Hospital, 'A Businco' Cagliari, Sardinia, Italy.*

**Summary** Postmenopausal patients with primary breast cancer were treated with tamoxifen, ethynyl-oestradiol or prednisolone for up to 12 days before mastectomy and the effects of pretreatments with these drugs on the activities of phosphofructokinase (PFK), 6-phosphogluconate dehydrogenase (6PGDH) and α-glycerolphosphate dehydrogenase (α-GPDH) in the carcinomas were compared with age, stage and menopausal status matched untreated controls. The administration of tamoxifen or prednisolone resulted in a significant increase in the activity of α-GPDH and the α-GPDH/6PGDH ratio, whereas ethynyl-oestradiol treatment produced a significant decrease in the activity of the enzyme and the ratio. When tamoxifen and ethynyl-oestradiol were administered together, it was found that tamoxifen failed to reverse the oestrogen-induced reduction in the activity of α-GPDH. Since increased activity of the enzyme or a higher α-GPDH/6PGDH ratio are associated with a lower risk of recurrence (Deshpande *et al.*, 1981), it is postulated that the beneficial effects of tamoxifen or prednisolone in terms of prolongation of the relapse free interval might be mediated via alterations in the activity of α-GPDH in micrometastases. The activities of PFK and 6PGDH remained unaffected by these treatments.

For the past eight years we have been exploring the usefulness of the measurements of the activities of certain enzymes of carbohydrate metabolism in primary carcinomas in prognosis in human breast cancer and have reported that higher activities of phosphofructokinase (PFK) and 6-phosphogluconate dehydrogenase (6-PGDH) or lower activity of α-glycerolphosphate dehydrogenase (α-GPDH) or lower α-GPDH/6PGDH ratios are associated with a high risk of recurrence (Deshpande *et al.*, 1981). Of the three enzymes, PFK is a key regulatory enzyme and the first enzyme unique to glycolysis. The other two enzymes are neither directly opposing nor key regulatory enzymes. They are involved in the channelling of substrates into the pathways of fat deposition (α-GPDH) or nucleic acid synthesis and production of reduced pyridine nucleotides (6PGDH). Yet their utility in predicting the likelihood of recurrence has been proven and therefore we are currently investigating whether the activities of these enzymes are amenable to manipulation. As a first step in this project we have examined the direct effects of tamoxifen, ethynyl-oestradiol (EE), prednisolone and various cytotoxic drugs, on the activities of these enzymes in MCF-7 cells in monolayer culture and observed that treatment of these cells with tamoxifen increases both the activity of α-GPDH and α-GPDH/6PGDH ratios whereas EE and prednisolone were without any effect. Of the cytotoxic drugs investigated so far, only adriamycin, at low doses, was found to consistently produce an increase in the activity of α-GPDH and α-GPDH/6PGDH ratios (Mitchell & Deshpande, 1984). In view of these findings we decided to investigate whether treatment of patients with these drugs before mastectomy would induce similar changes in the activities of these enzymes in the carcinomas. In this paper we report on the effects of pretreatment with tamoxifen, EE or prednisolone on the activities of these enzymes in primary carcinomas from a series of age- and stage-matched postmenopausal patients.

Correspondence: N. Deshpande.
Received 19 November 1984; and in revised form 11 April 1985.

### Materials and methods

### *Clinical*

The patients in this series of investigations were all postmenopausal women (age range 57–76) with carcinoma of the breast awaiting mastectomy. The presence of carcinoma was confirmed by histological examination of the tumour. For personal reasons some of them were unable to come to the hospital

© The Macmillan Press Ltd., 1985

for immediate treatment and therefore we took the opportunity to treat them with various drugs. The informed consent of each patient was obtained before the start of treatment prior to mastectomy. The patients were treated for between 7 and 12 days before mastectomy. The dosage of drugs was as follows: tamoxifen $2 \times 20\,mg\,day^{-1}$, EE $2\,mg\,day^{-1}$, prednisolone $3 \times 2.5\,mg\,day^{-1}$. The controls were 20 age, stage- and menopausal status-matched untreated patients. At mastectomy, a piece of carcinoma weighing ~400 mg was removed, wrapped in aluminium foil and frozen on solid $CO_2$. It was then transferred to the laboratory where it remained in liquid nitrogen until further processing.

### *Biochemical*

The tumours were partially thawed, cut into small pieces after removal of the surrounding fat, weighed and homogenized in a Silverson homogenizer in the medium described by Shonk & Boxer (1964). A small aliquot of each homogenate was kept for the estimation of DNA and the remainder of the homogenate was centrifuged in a refrigerated centrifuge (4°C, 800 *g*) for 20 min. The supernatant was decanted off, adjusted to a concentration of $50\,mg\,ml^{-1}$ and used as the source of enzymes. The activities of PFK, 6PGDH and α-GPDH were estimated according to the method of Shonk & Boxer (1964). The method of Burton (1956) was used for the estimation of DNA. The activities are expressed as $U\,mg^{-1}$ DNA where a unit is defined as that amount of enzyme which will catalyze the conversion of 1 μmol substrate $min^{-1}$. The results are expressed as mean ± s.d. for each group. The means were tested for statistical significance by Student's *t*-test.

### Results

The effects of the treatment of patients with tamoxifen, EE and both on the activities of PFK, 6PGDH, α-GPDH and α-GPDH/6PGDH ratios are presented in Table I. Tamoxifen treatment resulted in a significant increase in the activity of α-GPDH and α-GPDH/6PGDH ratios whereas EE treatment reduced the activity of the enzyme and the ratio. When patients were treated with a combined dose of the two drugs, the results show that EE was able to reverse the tamoxifen-induced increase in the activity of α-GPDH.

The effects of treatment with prednisolone are shown in Table II. The glucocorticoid treatment resulted in a significant increase in the activity of α-GPDH and α-GPDH/6PGDH ratios.

### Discussion

Since there are significant quantitative differences in the activities of certain enzymes in carcinomas between patients who are at a higher risk of recurrence and others in whom the risk is low, theoretically it should be possible to alter the enzyme patterns in the high risk group and investigate whether this results in the prolongation of the disease-free interval. In practice, it would mean inducing quantitative reductions in the

**Table I** Effects of ethynyl-oestradiol ($2\,mg\,day^{-1}$ for up to 12 days) and tamoxifen ($40\,mg\,day^{-1}$ for up to 12 days) treatments before mastectomy on the activities of PFK, 6PGDH, α-GPDH and α-GPDH/6PGDH ratios in human breast carcinomas. The results are expressed as units of enzyme activity $mg^{-1}$ DNA and are presented as mean ± s.d.

| | *Controls* (*n* = 20) | *Tamoxifen* (*n* = 9) | *Ethynyl-oestradiol* (*n* = 10) | *Ethynyl-oestradiol + tamoxifen* (*n* = 7) |
|---|---|---|---|---|
| PFK | 0.074 ± 0.043 | 0.075 ± 0.036 | 0.134 ± 0.082 | 0.072 ± 0.024 |
| 6PGDH | 0.073 ± 0.045 | 0.050 ± 0.031 | 0.074 + 0.021 | 0.054 ± 0.014 |
| α-GPDH | 0.078 ± 0.027 | 0.147 ± 0.083[a] | 0.045 ± 0.017[b] | 0.044 ± 0.020[c] |
| α-GPDH / 6PGDH | 0.95 ± 0.56 | 3.92 ± 2.69[a] | 0.48 ± 0.026[b] | 2.09 ± 2.04 |

[a]Significant differences between controls and tamoxifen treated patients $P < 0.01$.

[b]Significant differences between controls and ethynyl-oestradiol treated patients $P < 0.01$.

[c]Significant differences between tamoxifen and tamoxifen + ethynyl-oestradiol treated patients $P < 0.01$.

**Table II** Effects of prednisolone (2.5 mg three times $day^{-1}$ for up to 12 days) treatment before mastectomy on the activities of PFK, 6PGDH, α-GPDH and α-GPDH/6PGDH ratios in human breast carcinomas. The results are expressed as units of enzyme activity $mg^{-1}$ DNA and are presented as mean ± s.d.

| | *Controls* (*n = 20*) | *Prednisolone* (*n = 10*) |
|---|---|---|
| PFK | 0.074 ± 0.043 | 0.100 ± 0.042 |
| 6PGDH | 0.073 ± 0.045 | 0.070 ± 0.031 |
| α-GPDH | 0.078 ± 0.027 | 0.144 ± 0.079[a] |
| α-GPDH / 6PGDH | 0.95 ± 0.56 | 1.58 ± 0.74[a] |

[a]Significant differences between controls and prednisolone treated patients ($0.01 < P < 0.001$).

activities of PFK and 6PGDH and/or an increase in the activity of α-GPDH. As most drugs currently used in adjuvant therapies produce minor beneficial effects in terms of prolonged disease-free interval (Fisher *et al.*, 1975; Bonadonna *et al.*, 1976; Meakin *et al.*, 1979; Baum *et al.*, 1983; Goldhirsch *et al.*, 1984; Howell *et al.*, 1984), it was felt that our hypothesis might be more acceptable if it could be shown that at least some of these drugs act on carcinomas in such a way as to induce changes in activities of these enzymes. Therefore we have attempted to investigate the effects of two drugs, *viz.* tamoxifen and prednisolone, which are used in the endocrine treatments of these patients.

Tamoxifen is the most widely used drug in the treatment of endocrine responsive cancer. It was originally felt that its action on the carcinoma was mediated via the occupation of oestradiol binding sites in the tissue but recent studies indicate that it also antagonizes calmodulin (Lam, 1984), inhibits prostaglandin synthetase (Richie, 1978), suppresses plasminogen activator activity (Katzenellenbogen *et al.*, 1984), arrests the growth of cancer cells in the $G_0/G_1$ phase and induces a decline in the percentage of S-phase cells (Sutherland *et al.*, 1983).

The last finding indicates to us that the clinical observations of a prolonged disease-free interval in patients who received tamoxifen as an adjuvant to mastectomy might be due to such alterations to the growth rate of the carcinoma and that the finding of changes in cell cycle kinetics might be related to a reduction in the generation of energy for cell division. Since α-GPDH is the enzyme involved in the channelling of substrates into the pathways of fat deposition thereby depleting the amounts available for energy generation an increase in its activity both, in MCF-7 human breast cancer cells in monolayer culture (Mitchell & Deshpande, 1984) and in primary carcinomas from patients, after treatment with tamoxifen suggests that this might be associated with the retention of the malignant cells in the $G_0/G_1$ phase of the cell cycle which in turn produces inhibition of cell growth, leading to prolongation of the disease-free interval.

It is generally believed that a continuous oestrogenic stimulus is required to maintain the growth of breast carcinomas and that drugs which inhibit the action(s) of oestrogens will induce regressions in these tumours. In order to examine whether there is such an antagonism between oestrogens and tamoxifen in the activities of these enzymes, we have treated patients with tamoxifen, EE and tamoxifen plus EE. The data presented in Table I show that treatment with tamoxifen resulted in a significant increase and that with EE a significant decrease in the activity of α-GPDH and the α-GPDH/6PGDH ratio. This latter finding adds support to the hypothesis that a lower activity of the enzyme might be associated with a higher growth rate of the carcinoma. However, the data from patients treated with a combined dose of tamoxifen and EE suggest that EE is quite capable of reversing the tamoxifen-induced rise in the activity of the enzyme indicating that it is the oestrogen which determines the overall activity of the enzyme.

Prednisolone has been used as a treatment modality in both, endocrine and cytotoxic drug therapies (Meaking *et al.*, 1979, Goldhirsch *et al.*, 1984). Since the drug is rarely administered alone, it is difficult to judge whether it can act alone or only in association with other treatments such as ovarian irradiation or cytotoxic drugs. The data reported in Table II indicate that like tamoxifen, it is capable of increasing the activity of α-GPDH and α-GPDH/6PGDH ratios. Since these two drugs have different biological activities, the similarity in their action on the activity of α-GPDH suggests that either they act by binding to specific individual receptors or there might be a common focus involved in the regulation of the activity of the enzyme in breast carcinomas. It would be interesting to investigate other factors which increase the activity of the enzyme and to assess their usefulness clinically in patients who are failing to respond to tamoxifen or prednisolone.

In conclusion, these investigations indicate that both tamoxifen and prednisolone which are used in adjuvant endocrine therapies might be acting on the carcinoma in such a way as to induce alteration in the activity of α-GPDH which might be associated with changes in the growth of the carcinoma. We are currently extending these studies to investigate the role of certain cytotoxic drugs on the activities of the enzymes.

The authors are indebted to Drs R.D. Bulbrook and R.D. Rubens for their help in the preparation of the manuscript.

### References

BAUM, M., BRINKLEY, D.M., DOSSETT, J.A., PATTERSON, J.S., SMIDDY, F.G., WILSON, A. & 6 others. (1983). Improved survival amongst patients treated with adjuvant tamoxifen after mastectomy for early breast cancer. *Lancet*, **ii**, 450.

BONADONNA, G., BRUSAMOLINO, E., VALAGUSA, P. & 8 others. (1976). Combination chemotherapy as an adjuvant treatment in operable breast cancer. *N. Engl. J. Med.*, **294**, 405.

BURTON, K. (1956). A study of the conditions and mechanism of the diphenylamine reaction for the colorimetric estimation of deoxyribonucleic acid. *Biochem. J.*, **62**, 315.

DESHPANDE, N., MITCHELL, I. & MILLIS, R. (1981). Tumour enzymes and prognosis in human breast cancer. *Eur. J. Cancer*, **17**, 443.

FISHER, B., CARBONE, P., ECONOMOU, S.G. & 7 others. (1975). L-Phenylalanine mustard (L-Pam) in the management of primary breast cancer. *N. Engl. J. Med.*, **292**, 117.

GOLDHIRSCH, A., STJERNSWARD, J. & others. (Ludwig Breast Cancer Study Group) (1984). Randomized trial of chemo-endocrine therapy and mastectomy alone in post-menopausal patients with operable breast cancer and axillary node metastasis. *Lancet*, **i**, 1256.

HOWELL, A., GEORGE, W.D., CROWTHER, D. & 8 others. (1984). Controlled trial of adjuvant chemotherapy with cyclophosphamide, methotrexate and fluorouracil for breast cancer. *Lancer*, **ii**, 307.

KATZENELLENBOGEN, B.S., NORMAN, M.J., ECKERT, R.L., PELTZ, S.W. & MANGEL, W.F. (1984). Bioactivities, estrogen receptor interactions and plasminogen activator-inducing activities of tamoxifen, hydroxytamoxifen isomers in MCF-7 human breast cancer cells. *Cancer Res.*, **44**, 112.

LAM, H.Y.P. (1984). Tamoxifen is a calmodulin antagonist in the activation of C-AMP phosphodiestrase. *Biochem. Biophys. Res. Commun.*, **118**, 27.

MEAKIN, J.W., ALLT, W.E.C., BEALE, F.A. & 11 others. (1979). Ovarian irradiation and prednisone therapy following surgery and radiotherapy for carcinoma of the breast. *Canad. Med. Assoc. J.*, **120**, 1221.

MITCHELL, I. & DESHPANDE, N. (1984). Drug effects on certain enzymes of carbohydrate metabolism in MCF-7 cells in culture. *Clin. Oncol.*, **10**, 253.

RICHIE, G. (1978). The direct inhibition of prostaglandin synthetase of human breast cancer tissue by "Nolvadex". *Rev. Endocrine-related Cancer*, **35**, (suppl.).

SHONK, C.E. & BOXER, G.E. (1964). Enzyme patterns in human tissues. I. Methods for the estimation of glycolytic enzymes. *Cancer Res.*, **24**, 709.

SUTHERLAND, R.L., HALL, R.E. & TAYLOR, I.W. (1983). Cell proliferation kinetics of MCF-7 human mammary carcinoma cells in culture and effects of tamoxifen on exponentially growing and plateau phase cells. *Cancer Res.*, **43**, 3998.